# Hourly emissions of air pollutants and greenhouse gases from open biomass burning in China during 2016–2020

**DOI:** 10.1038/s41597-023-02541-0

**Published:** 2023-09-16

**Authors:** Yuanqian Xu, Zhijiong Huang, Jiashu Ye, Junyu Zheng

**Affiliations:** 1 School of Materials and Chemical Engineering, Zhengzhou University of Light Industry, Zhengzhou, 450001 China; 2 Institute for Environment and Climate Research, Jinan University, Guangzhou, 511443 China; 3 Sustainable Energy and Environment Thrust, The Hong Kong University of Science and Technology (Guangzhou), Guangzhou, 511453 China

**Keywords:** Atmospheric chemistry, Atmospheric chemistry

## Abstract

Open biomass burning (OBB) is a significant source of air pollutants and greenhouse gases that have contributed to air pollution episodes in China in recent years. An accurate emission inventory is critical for the precise control of OBB. Existing OBB emission datasets are commonly based on MODIS observations, and most only have a daily-scale temporal resolution. Daily OBB emissions, however, might not accurately represent diurnal variations, peak hours, or any open burning processes. The China Hourly Open Biomass Burning Emissions (CHOBE) dataset for mainland China from 2016 to 2020 was developed in this study using the spatiotemporal fusion of multiple active fires from MODIS, VIIRS S-NPP and Himawari-8 AHI detections. At a spatial resolution of 2 km, CHOBE provided gridded CO, NOx, SO_2_, NH_3_, VOCs, PM_2.5_, CO_2_, CH_4_ and N_2_O emissions from OBB. CHOBE will enhance insight into OBB spatiotemporal variability, improves air quality and climate modelling and forecasting, and aids in the formulation of precise OBB preventive and control measures.

## Background & Summary

Open biomass burning (OBB) typically involves forest, grassland and in-field crop straw fires. In China, over 95% of the total OBB emissions are accounted for by the in-field crop straw and forest fires^1,2^. The disposal of crop straw increasingly shifted from indoor combustion to in-field burning as China’s economy developed^3^. As a result, the crop straw that would otherwise be burned in a dispersed manner is burned in a concentrated time and place, causing China to experience severe haze periods and detrimental health effects^4–11^. On the other hand, OBB emissions increased by more than 50% from 2003 to 2014, and have fluctuated at the national scale in China in recent years^2,3^. Additionally, OBB emissions were a substantial source of air pollutants and greenhouse gases in China, contributing 20% and 6% of the national CO and CO_2_ emissions, respectively, in 2017^12–14^.

A high-resolution OBB emission inventory is necessary for OBB regulation and atmospheric simulations. Globally, several OBB emission datasets, including GFED^15,16^, GFAS^17^, FINN^18^ and FEER^19^, had been developed. In addition, there are several national OBB emissions datasets in China based on Moderate Resolution Imaging Spectroradiometer (MODIS) fire point/burned area observations^2,20–22^ or crop yields^1,23^. However, due to the significant uncertainty in the activity data used to calculate emissions, these OBB emission datasets still have limitations. First, OBB emissions in China tend to be underestimated due to the coarse spatial resolutions of MODIS (1 km for active fires and 500 m for burned areas) which cannot detect tiny fire occurrences^3,24^. Second, some studies used prescribed spatial and temporal profiles based on MODIS observations or land-use to allocate city-based/county-based emissions estimated using the crop yields-based method to get a model-ready dataset for air quality simulations^23,25^. but these prescribed profiles cannot reflect the dynamic variations of OBB emissions^26–28^. Third, the hourly variation of OBB emissions cannot be resolved by the most commonly used MODIS datasets, which only observe four times at local 01:30, 10:30, 13:30 and 22:30^29^.

To address the aforementioned restrictions, we developed an hourly OBB emission dataset from 2016 to 2020 in mainland China, which is named the China Hourly Open Biomass Burning Emissions (CHOBE), using a newly developed OBB emission estimation approach^30^. The high-resolution activity data of CHOBE benefit from the spatiotemporal fusion of multiple active fires detected by MODIS, the Visible Infrared Imaging Radiometer onboard the Suomi National Polar-orbiting Partnership (VIIRS S-NPP) and Himawari-8 AHI. 6 air pollutants (CO, NOx, SO_2_, NH_3_, VOCs, PM_2.5_) and 3 greenhouse gases (CO_2_, CH_4_, N_2_O) are included in CHOBE, which can provide hourly data supporting air quality and climate simulations and OBB control.

## Methods

### Dataset and scopes

In-field crop straw burning and forest fires are the main source sectors in this study since they are responsible for more than 95% of China’s OBB emissions^1,2^. Due to its high fuel loading per unit area, forest fire emissions are characterized by small emission density, high intensity and long duration, whereas crop straw burning emissions have wider spatial distribution, low intensity and short duration^2,29^. Hence, crop straw burning and forest fire emissions were estimated separately.

In addition to the commonly used MODIS observations, most of the small active fires that MODIS have missed could be captured by using VIIRS S-NPP active fires, which have a higher spatial resolution of 375 m and a monitoring frequency of twice per day (at 01:30 and 13:30 local time). The temporal resolution of OBB emission was resolved to an hourly scale using Himawari-8 AHI observations, which have a temporal resolution of 10 min and a spatial resolution of 2 km^29–31^. MODIS and VIIRS S-NPP active fires were obtained from the Fire Information for Resource Management System (FIRMS) of NASA (https://firms.modaps.eosdis.nasa.gov/)^32^, and Himawari-8 AHI active fires were from the P-Tree System of Japan Aerospace Exploration Agency (JAXA) (https://www.eorc.jaxa.jp/ptree/)^33^.

**Fig. 1:**
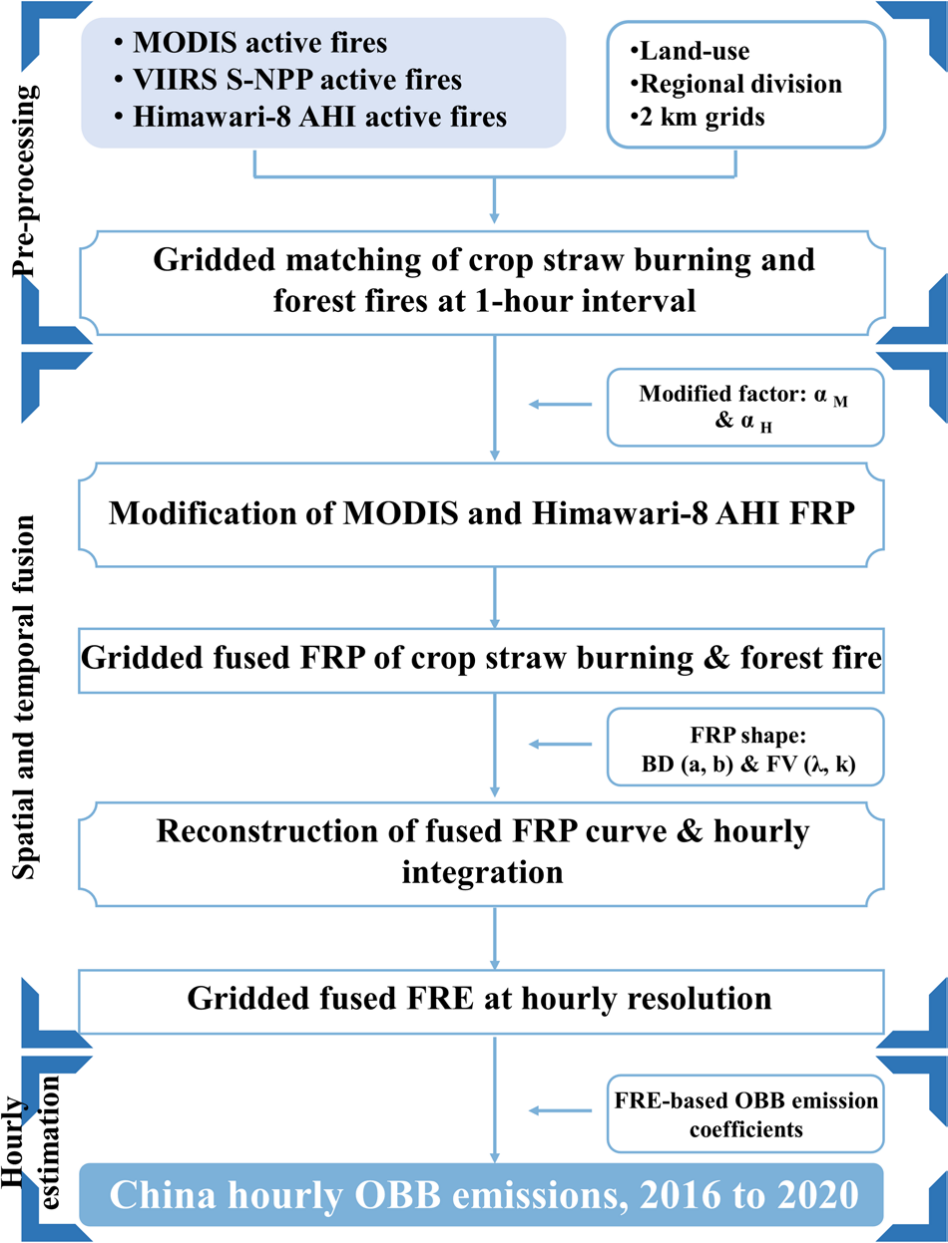
Flowchart of the hourly OBB emission estimation.

To estimate OBB emissions across mainland China from 2016 to 2020, multiple active fires observed by MODIS, VIIRS S-NPP and Himawari-8 AHI were pre-processed. As shown in Fig. 1, the estimation of hourly gridded OBB emissions involves three steps: 1) Pre-processing of multiple active fires; 2) Spatial and temporal fusion of multiple active fires from polar-orbiting and geostationary satellite observations to achieve hourly OBB activity data and 3) Regional estimation of OBB emission coefficients and hourly estimation of gridded emissions. The hourly OBB emissions of CO, NOx, SO_2_, NH_3_, VOCs, PM_2.5_, CO_2_, CH_4_, N_2_O from 2016 to 2020 were spatially distributed into 2.41 × 10^6^ grids with a spatial resolution of 2 km.

### Pre-processing of multiple active fires

Since fire pixels with low-confidence were typically treated as clear, non-fire or land pixels, active fires with high confidence (MODIS ≥ 30%, VIIRS S-NPP is N or H, and Himawari-8 AHI ≥ 2) were employed to confirm the accuracy of emission estimates^34^. A total of 3.05 × 10^5^ MODIS active fires, 1.43 × 10^6^ VIIRS S-NPP active fires and 4.54 × 10^6^ Himawari-8 AHI active fires were applied. Based on the spatial patterns of agriculture and forest land-use, these active fires were categorized into crop straw burning and forest fires.

Our previous research demonstrated that there are considerable regional discrepancies in spatiotemporal variations and driving forces of OBB emissions^35^. We classified mainland China into 7 crop straw burning regions according to China’s provincial agricultural habits and boundaries (Supplementary Table 1), and 4 forest fires regions based on the geographical distributions of forest types (as shown in Supplementary Fig. 1) followed by our previous study^30^. Then, crop straw burning and forest fires were assigned to these 7 crop straw burning regions and 4 forest fire regions based on their latitude and longitude.

Crop straw burning and forest fires were separately matched to the 2 km grids using a 1-hour time step by assuming that there is little change in the fire radiative power (FRP) variation during OBB within an hour to identify the statistical relationships of multiple active fires derived from various satellites. If there is more than 1 active fire from MODIS or VIIRS S-NPP at the same time, it is considered that multiple small fires occur simultaneously in a 2 km grid and their FRP is added. Regarding Himawari-8 AHI detection, its 6 detections of FRP within an hour are averaged. As a result, by the matching multiple active fires, OBB detections are transformed from point-by-point to grid-based observations (referred to as grided FRP), and crop straw burning and forest fires would be converted to gridded FRP separately if they are on the same grid.

### Spatial and temporal fusion of multiple active fires

According to our previous study^30^, the parameters in Table 1 were used to fuse multiple active fires. To solve the problem of FRP underestimation of MODIS and Himawari-8 AHI observations caused by their coarse spatial resolution, modified factor α was gained from the relationships between contemporaneous VIIRS-MODIS and VIIRS-Himawiri-8 AHI gridded FRP (see Supplementary Table 2) considering that VIIRS S-NPP FRP was more reflective of OBB than MODIS and Himawari-8 AHI active fires due to its high spatial resolution^36,37^. The gridded FRP derived from MODIS and Himawari-8 AHI was modified by multiplying α.

**Table 1:** Parameters for the spatiotemporal fusion of multiple active fires.

Parameter	Usage	Source
α	FRP modification factor of MODIS fires and Himawari-8 AHI first for different regions	See Supplementary Table 2
BD (a, b)	Parameters for burning duration calculation for different regions	See our previous study Table S1^30^
FRP variations (λ, k)	Scale and shape parameters of Weibull distribution in the FRP variations for different regions and BD	See our previous study Table S2^30^

It is necessary to screen the contemporaneous gridded FRP since the overlaps of overpassing time from various satellites. The principle of choosing the high spatial resolution of contemporaneous active fires was used to screen the overlapped gridded FRP from VIIRS S-NPP, modified MODIS and Himwari-8 AHI. Hence, the hourly fused FRP was calculated by using Eq. (1).

1 $$FR{P}_{fused}={w}_{1}\times FR{P}_{Hm}+{w}_{2}\times FR{P}_{Mm}+{w}_{3}\times FR{P}_{V}$$

where *FRP*_*fused*_ standards for the gridded FRP within an 1-hour bin; *FRP*_*Hm*_ and *FRP*_*Mm*_ represent the modified Himawari-8 AHI and MODIS FRP, respectively; *FRP*_*V*_ is the VIIRS S-NPP FRP; *w*_1_, *w*_2_, and *w*_3_ are the fusion weights in which *w*_3_=1, *w*_1_=0 and *w*_2_=0 if *FRP*_*V*_ was available, *w*_2_=1, *w*_1_=0 and *w*_3_=0 if *FRP*_*Mm*_ was valid and *FRP*_*V*_ was unavailable, *w*_1_=1, *w*_2_=0 and *w*_3_=0 if only *FRP*_*Hm*_ was valid.

Our previous study revealed that changes in burning duration (BD) and FRP variation during the OBB combustion cycle is closely related to the observed FRP values^30^. Thus, BD was determined by 11 regression models as shown in Supplementary Fig. 2 and Eq. (2), which were fitted by Himawari-8 AHI observed fire cycles from 2016 to 2020.

2 $$BD={a}_{r}\times FR{P}_{p}+{b}_{r}$$

where *a*_*r*_ and *b*_*r*_ are the parameters of the regression models for 7 crop straw burning and 4 forest fire regions (r). *FRP*_*p*_ stands for the peak FRP during a fire cycle.

FRP variations were determined by 86 Weibull curves as a function of peak FRP while the related BD was larger than 4 hours (Eq. 3), which was fitted by historical observations of burning cycles from Himawari-8 AHI for each region (see Supplementary Fig. 3). Otherwise, FRP was regarded as Gaussian distributions followed by Vermote *et al*.^27^ since small fires with short BD cannot be effectively detected by Himawari-8 AHI (Eq. 4). According to the fused FRP and calculated BD, Weibull curves was adopted for 34% of the crop straw burning fires and 37% of forest fires.

3 $$FRP\left(t\right)=FR{P}_{p}\times {\left(t/{t}_{m}\right)}^{k-1}\times \frac{{e}^{{\left({t}_{m}/\lambda \right)}^{k}}}{{e}^{{\left(t/\lambda \right)}^{k}}}$$

where t is the burning time from the beginning of a fire cycle. *t*_*m*_ represents the period before the peak FRP during the fire cycle. λ and k stand for the scale and shape parameters of the Weibull distribution, which were fitted for each BD in different regions.

4 $$FRP\left(t\right)=FR{P}_{p}\times \left[{e}^{-\frac{{\left(t-\mu \right)}^{2}}{2\times {\sigma }^{2}}}\right]$$

Where *μ* and *σ* denote to parameters of the Gaussian distribution, *μ* is the middle time of BD and *σ* is quarter of BD.

Moreover, we judged the continuity for adjacent fused FRP based on their values and variations to prevent the repeated integration of fused FRP that may belong to a continuous OBB combustion process. They were regarded as the same OBB event if the latter FRP is within ± 60% range of the change curve of the previous fused FRP. and the FRP change curve was redraft with partial retention before the vertex of the first fused FRP curve, a linear change between continuous fused FRP and Partial retention after the vertex of the last fused FRP curve.

By hourly integrating the constructed FRP curves, gridded fire radiative energy (FRE) was estimated to produce high-resolution OBB activity data.

### Hourly estimation of gridded OBB emissions

To obtain greenhouse gas emission datasets from OBB, FRE-based emission coefficients of CO_2_, CH_4_ and N_2_O were also estimated by following the method shown in our previous study^30^. 21 regression models of regional fused FRE and historical emissions based on statistical data were built to fit the FRE-based crop straw burning emission coefficients of CO_2_, CH_4_, N_2_O (see Table 2). In terms of forest fires, their FRE-based emission coefficients were quantified by multiplying the local dry matter-based emission coefficients and conversion factor from FRE to dry matter consumption (0.41 kg/MJ)^27^.

**Table 2:** Regional FRE-based OBB emission coefficients of greenhouse gases in g/MJ.

Region	CO_2_	CH_4_	N_2_O
In-field crop straw burning Region 1	784.18	2.51	0.04
In-field crop straw burning Region 2	877.97	2.64	0.04
In-field crop straw burning Region 3	1060.30	3.07	0.05
In-field crop straw burning Region 4	870.50	2.60	0.05
In-field crop straw burning Region 5	863.48	2.73	0.05
In-field crop straw burning Region 6	697.63	2.22	0.04
In-field crop straw burning Region 7	747.28	2.24	0.04
Evergreen broad-leaf forest fires	673.63	2.09	0.11
Mixed forest fires	668.30	2.05	0.11
Deciduous broad-leaf forest fires	668.30	2.05	0.11
Needle-leaf forest fires	620.74	2.46	0.11

The gridded OBB emissions were estimated using Eq. (5) based on the fused hourly FRE and regional FRE-based OBB emission coefficients.

5 $${E}_{p}={\sum }_{r=1}^{7}\left(FR{E}_{CB,r}\times E{C}_{CB,r,p}\right)+{\sum }_{s=1}^{4}\left(FR{E}_{FF,s}\times E{C}_{FF,s,p}\right)$$

where *E*_*p*_ refers to the grided OBB emission of atmospheric component *p*; *FRE*_*CB,r*_ stands for the gridded crop straw burning FRE at hourly resolution in crop straw burning region *r*; *EC*_*CB,r,p*_ represents FRE-based crop straw burning emission coefficients in crop straw burning region *r* of atmospheric component *p*; *FRE*_*FF,s*_ is the grided forest fire FRE at hourly resolution in forest fire region *s*; *EC*_*FF,s,p*_ is the FRE-based forest fire emission coefficient.

## Data Records

The CHOBE datasets can be found at figshare^38^. A total of 19 hourly data records (coordinates of 2 km grids and hourly emission inventories) are contained in the datasets. Of those,

- 1 is the central longitude and latitude of the 2 km-grids across mainland China used in this study [file “China 2 km-grids longitude and latitude”];
- 9 are the hourly gridded OBB emissions in mainland China (2016–2020) for different types of air pollutants and greenhouse gases [file “Hourly gridded ‘*type*’ emissions, 2016–2020”];
- 9 are the hourly provincial OBB emissions in mainland China (2016–2020) for different types of air pollutants and greenhouse gases [file “Hourly provincial ‘*type*’ emissions, 2016–2020”].

The hourly OBB gridded emission inventories were tabulated in a uniform table with 4.02 × 10^6^ rows and 26 columns. The 4.02 × 10^6^ rows represent each grid with OBB emission that occurred from 2016 to 2020. The 26 columns are (1) the first column is the grid ID, with their corresponding central longitude and latitude shown in the file “China 2 km-grids longitude and latitude”; (2) the second column is the date of OBB emissions; (3) the 3–26 columns are hourly gridded OBB emissions in gram from 00:00 to 23:59 during the day, respectively. The provincial OBB emission inventories are tables with 52288 rows and 26 columns. The 52288 rows stand for OBB emissions in different provinces from 2016 to 2020, and the columns are hourly OBB emissions for the related province.

**Fig. 2:**
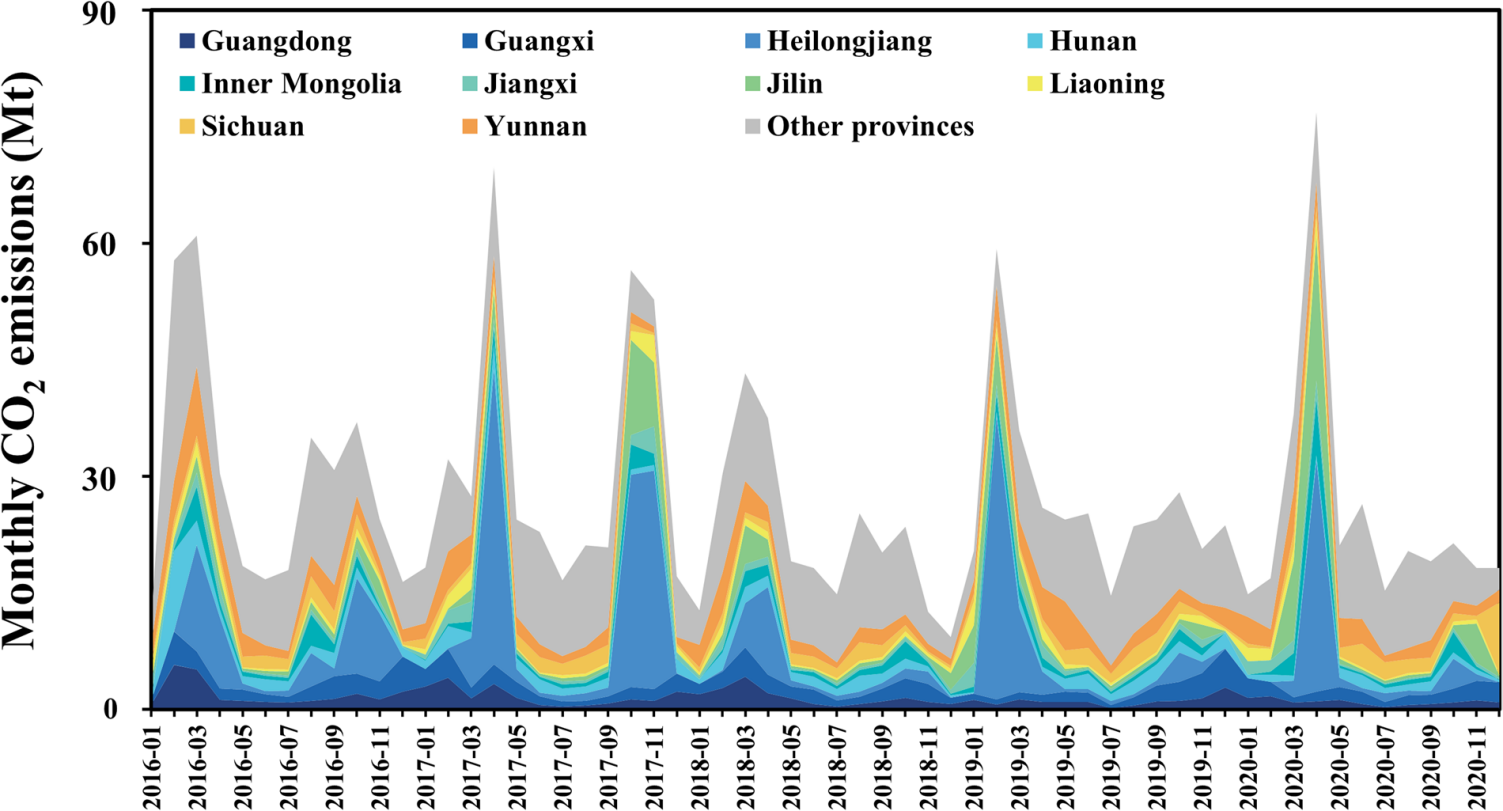
Monthly CO_2_ emissions from OBB in mainland China from 2016 to 2020 with the top 10 provinces noted.

Table 3 presents annual emissions of CO, NOx, SO_2_, NH_3_, VOCs, PM_2.5_, CO_2_, CH_4_, N_2_O from 2016 to 2020 in mainland China. For how these estimates compare to other OBB emission datasets, please see the Technical Validation section. Figure 2 shows the monthly CO_2_ emissions in mainland China with the top 10 provinces noted from 2016 to 2020 and annual CO_2_ emissions in the 31 provinces were shown in Supplementary Table 3. Annual OBB emissions from 2016 to 2020 in mainland China decreased with fluctuations. The top 10 (out of 31 total) provinces typically contributed more than 80% of the national OBB emissions in the peak months of February, March, and April, while in other months they only made up about 50% of China’s total OBB emissions. Particularly in Heilongjiang, OBB emissions from the top 10 provinces are the main areas contributing to the peaks.

**Table 3:** Annual OBB emissions in mainland China from 2016 to 2020 in Mt.

Component	2016	2017	2018	2019	2020
CO	18.10	18.80	13.50	16.20	15.20
NOx	0.73	0.83	0.54	0.69	0.66
SO_2_	0.13	0.15	0.10	0.123	0.12
NH_3_	0.22	0.24	0.17	0.20	0.19
VOCs	3.43	3.35	2.53	2.90	2.74
PM_2.5_	2.54	2.80	1.88	2.35	2.23
CO_2_	357	380	266	326	306
CH_4_	1.11	1.19	0.83	1.02	0.96
N_2_O	0.04	0.04	0.03	0.03	0.03

## Technical Validation

### Spatial coverage

**Fig. 3:**
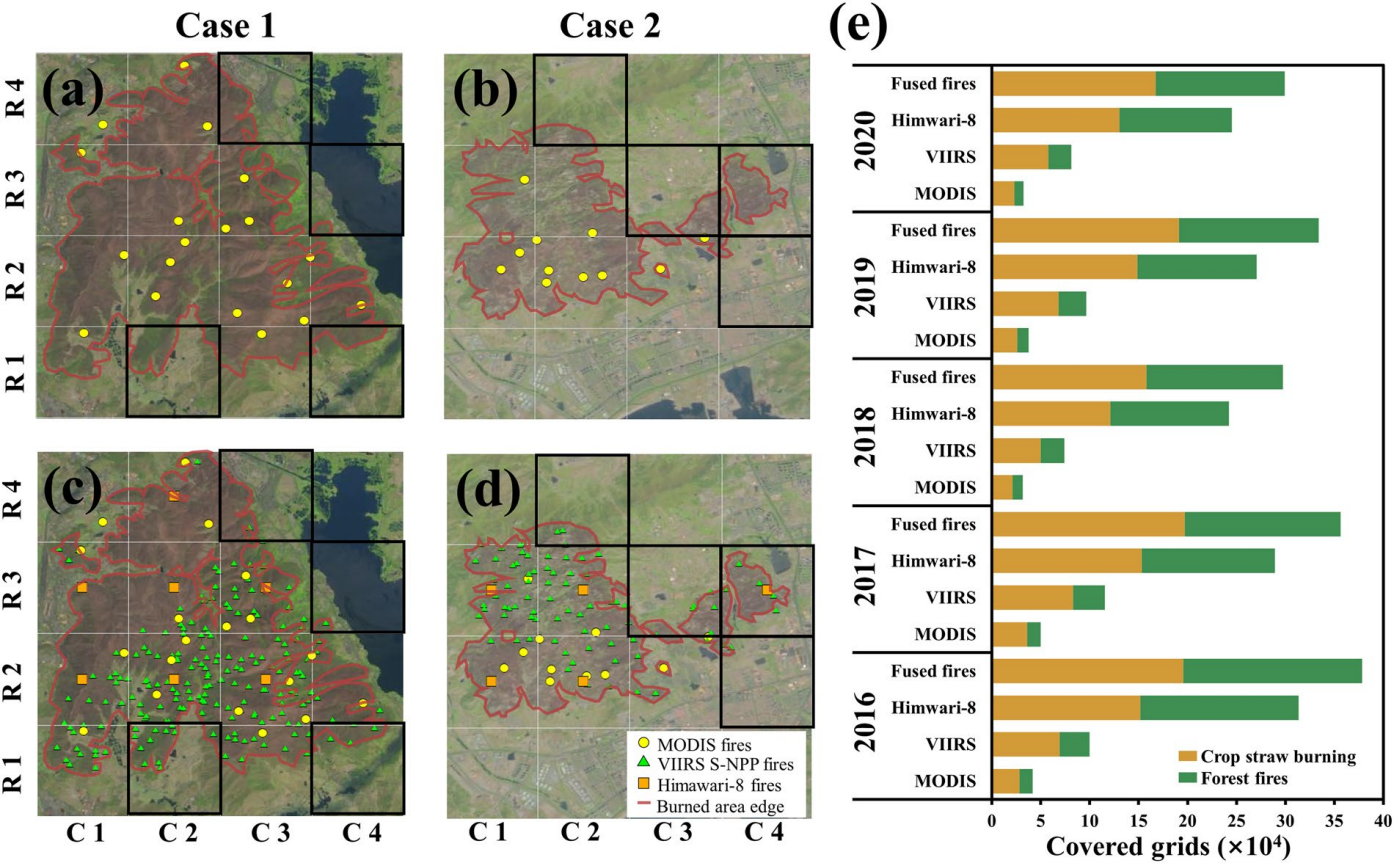
Spatial distributions of MODIS, VIIRS S-NPP and Himwari-8 active fires during typical cases (**a**–**d**) and comparisons of covered 2 km-grids number of different active fires from 2016 to 2020 (**e**). Case 1: a & c, Xichang forest fires from March 30 to April 2, 2020 in Sichuan province. Case 2: b & d, Qingdao forest fires from April 23 to April 25, 2020 in Shandong province. The true color images highlighted the burned area’s edge in red, the 2 km-grid was in grey, and the grids with OBB that were missed by MODIS active fires were in black.

**Fig. 4:**
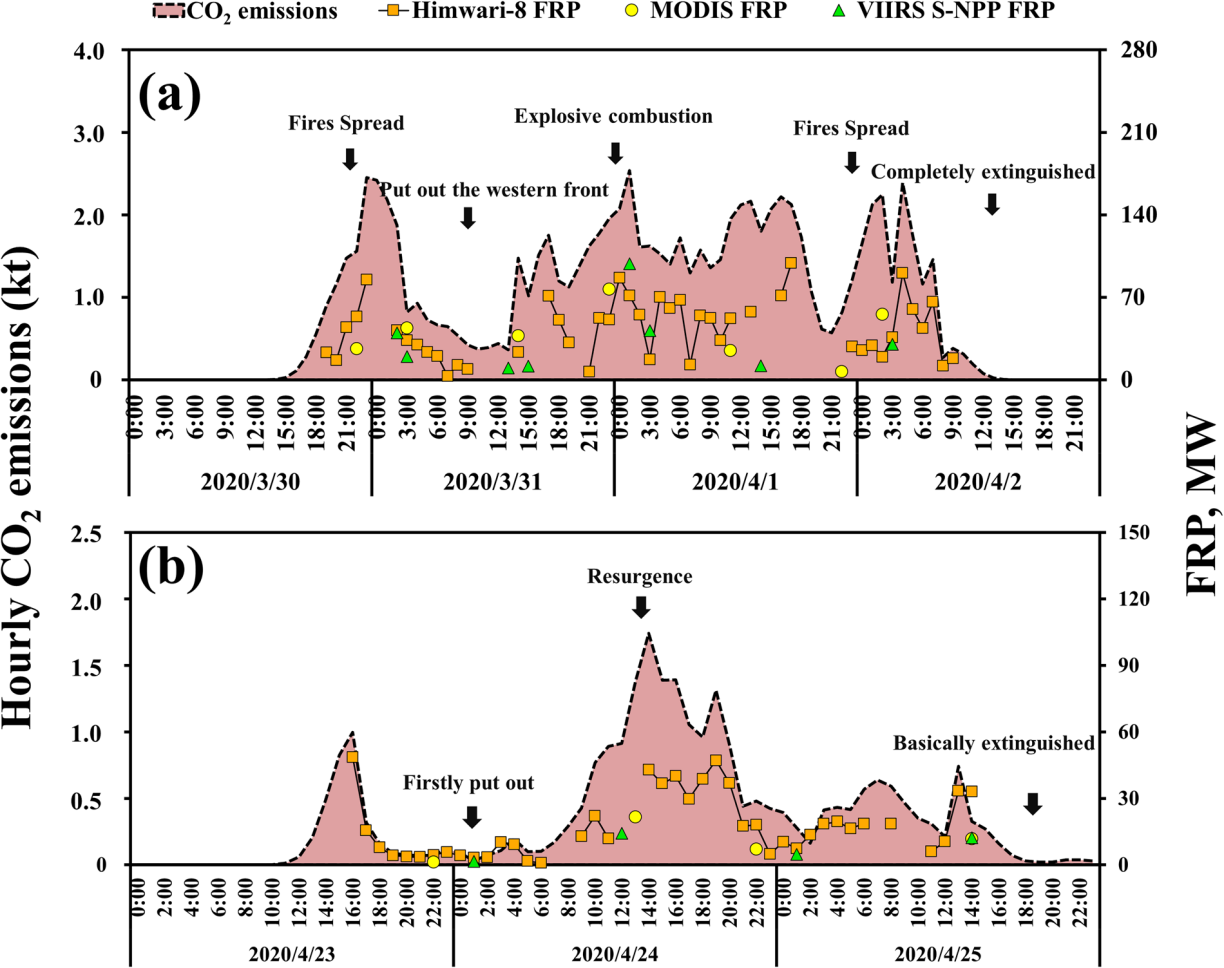
Hourly FRP from different satellite observations and OBB emissions estimated in this study during two typical cases as presented in Fig. 3.

Using the Sichuan Xichang (Case 1, from March 30 to April 2, 2020) and Shandong Qingdao (Case 2, from April 23 to April 25, 2020) forest fires as examples, we exhibited the allocations of MODIS, VIIRS S-NPP and Himawari-8 AHI active fires to explore the differences in spatial coverage of the multiple active fires and purely MODIS active fires (Fig. 3a–d). There are 4 grids in Case 1 (Fig. 3a) and 4 grids in Case 2 (Fig. 3b) with OBB that were missed by MODIS active fires. The deficiencies are made up by VIIRS-SNPP and Himawari-8 AHI active fires, as indicated by the black frame. Given that multiple active fires have complete spatial coverage, it is possible that the fused fires employed in this study can capture more burned grids than the active fires typically used by MODIS.

At the national scale, as shown in Fig. 3e, the ratio of VIIRS S-NPP to MODIS-covered grids was 2.2~2.7 for both crop straw burning and forest fires due to the high spatial resolution of VIIRS S-NPP. Since the high temporal resolution, Himawari-8 AHI covered grids were enhanced by around 5 times and 10 times for crop straw burning and forest fires, respectively, compared with MODIS. As a result, the average annual number of 2 km grids covered by fused OBB fires was 8.7 times higher than what was observed by MODIS, indicating that more OBB was covered by fused fires.

### Temporal fluctuations

Hourly FRP from various satellite observations and OBB emissions during two typical OBB cases (same as Fig. 3) were shown in Fig. 4 to further illustrate the temporal changes of FRP during a fire event. Neither polar-orbiting satellites nor geostationary satellite FRP can continually capture the variations of forest fire activity. Hourly variations during the period were vividly portrayed by reconstructing the FRP variations using the BD models and Weibull curves. In Case 1, CHOBE successfully recorded the news’ reported propagation of the open fires at night on April 1. The temporal alterations in Case 2 were likewise accurately captured by CHOBE, particularly for the resurgence on April 24 at noon. In Case 2, CHOBE also accurately captured the temporal changes, especially for the resurgence at noon on April 24. The consistency between the temporal variations reflected by CHOBE and news reports illustrates its ability to capture the hourly changes in OBB emissions.

### Uncertainties

Uncertainties of OBB emission estimates mainly derive from activity data and emission factors^24,39^. In this study, hourly fused FRE was used as the activity data to estimate hourly OBB emissions. Uncertainties in fused FRE mainly come from the observed FRP, BD calculation and the fused FRP variations. According to Freeborn *et al*., the variability of MODIS FRP was ±53.2% for a single active fire, but it can decrease to less than ± 5% when more than 50 active fires were aggregated^40^. Thus, we assumed the coefficient of variation (CV) observed FRP from multiple satellites was about 5% since only active fires with high confidence were employed, and MODIS and VIIRS active fires were both aggregated to 2 km grids. The comparison between observed BD and predicted BD based on fitting parameters of *a* and *b* revealed that the CV of BD was about 40%. Also, according to the gaps in observed FRP variations and predicted FRP variations, the CV of fused FRP variations was about 10%. It was assumed that these parameters used to quantify the hourly active data follow a normal distribution.

The uncertainties of the gridded daily FRE for each region were displayed in Supplementary Table 4 in accordance with the Monte Carlo approach. Uncertainties in gridded daily FRE ranged from −25% to 45% in most regions, whereases crop straw burning Region 4 has the largest uncertainties, ranging from −67% to 78%. The following factors contributed to the high uncertainty in crop straw burning Region 4: (1) its correlation coefficient (R^2^) of BD fitting was 0.7521, which is lower than in other regions. (2) FRP curve is determined by BD, FRP and fires continuity. The FRP curve of independent fires (i. e., no fires were observed in 1 hour before and after at the same grid) is drawn in accordance with BD, but the FRP between continuous fused fires is thought to be linear. As a result, FRP curve of independent fires typically has larger uncertainty. The proportion of independent fires in crop straw burning Region 4 was 75%, but proportions in other regions ranged from 43% to 69%, indicating that more FRP curves were classified as separate OBB event, with larger uncertainties.

As presented in Supplementary Table 5, the FRE-based crop straw burning and forest fire emission coefficients generated by the statistical model and coefficient conversion, respectively, were comparable to prior studies. the uncertainties of the FRE-based OBB emission coefficients applied in this study were difficult to measure due to the inability to ascertain the uncertainty of specific parameters, such as the uncertainties of regression models of regional fused FRE and historical emissions based on statistical data. The CVs of FRE-based crop straw burning and forest fire emission coefficients were thus obtained from Andreae^41^ and are shown in Table 4 under the presumption that 7 crop straw burning regions and 4 forest fire regions share the same CV of emission coefficients.

**Table 4:** CVs of crop straw burning and forest fires emission coefficients.

Component	Crop straw burning	Forest fires
CO	72%	44%
NOx	51%	59%
SO_2_	88%	68%
NH_3_	63%	71%
VOCs	105%	88%
PM_2.5_	54%	78%
CO_2_	16%	8%
CH_4_	104%	53%
N_2_O	43%	46%

By applying the Monte Carlo method with 100,000 simulations, we quantified the uncertainties of the annual OBB emissions associated with activity data and emission coefficients. The normal distribution was assumed for both activity data and emission coefficients with the CVs mentioned above. Supplementary Table 6 displays OBB emission uncertainties with 95% confidence intervals for each region. Due to the large uncertainty in FRE, crop straw burning Region 4 experienced slightly larger uncertainty than in the other regions. The diversity in uncertainties of crop straw burning emission and forest fire were primarily responsible for the variances in FRE-based emission coefficients. The uncertainty range of annual OBB emissions in mainland China were −45%~45% for CO emissions, −44%~ 44% for NOx emissions, −64% ~ 65% for SO_2_ emissions, −53% ~ 53% for NH_3_ emissions, −75% ~ 76% for VOCs emissions, −56% ~ 56% for PM_2.5_ emissions, −10% ~ 11% for CO_2_ emissions, −70% ~ 71% for CH_4_ emissions and −39% ~ 40% for N_2_O emissions.

### Comparison with existing datasets

**Fig. 5:**
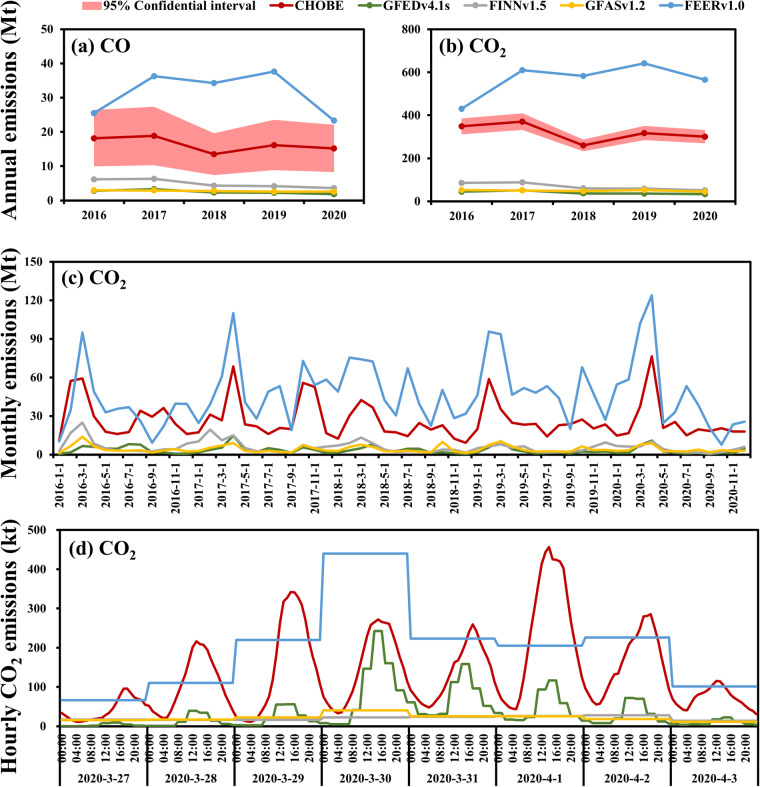
Comparisons with existing datasets of annual OBB CO (**a**) and CO_2_ (**b**) emissions, monthly OBB CO_2_ (**c**) emissions and hourly CO_2_ (**d**) emissions during a high emitted period from 27 March to 3 April, 2020, in mainland China. The hourly GFEDv4.1 s emissions were calculated by taking the average of the 3-hourly emissions. The hourly emissions from FINNv1.5, GFASv1.2 and FEERv1.0 were obtained by dividing the daily emissions by 24 hours since they have a temporal resolution of one day.

Mainland China’s annual, monthly and hourly (during a high emitted period from 27 March to 3 April, 2020) OBB estimates in CHOBE were compared to existing global OBB emission datasets (Fig. 5) to validate the CHOBE datasets established in this study. Similar to Pan *et al*.^39^, we also discover significant gaps between several OBB emission datasets. Compared with GFEDv4.1 s (https://www.globalfiredata.org/data.html)^16^, FINNv1.5 (https://www.acom.ucar.edu/Data/fire/)^18^ and GFASv1.2 (https://www.ecmwf.int/en/forecasts/dataset/global-fire-assimilation-system)^17^, the annual OBB emissions in CHOBE are 2~7 times higher, and the monthly emissions are 1~48 times higher. This is expected because these datasets, which all used MODIS observations, cannot observe some fire events due to the coarse resolution of 1 km and for active fires and 500 m for burned areas and the low monitor frequency of 4 times per day. Compared with FEER v1.0 (https://feer.gsfc.nasa.gov/data/emissions/)^19^, CHOBE emissions are lower by about 40%. This is because FEER applied the MODIS AOD constrained emission coefficients, which were greatly overestimated in China and about 10 times higher than the OBB emission coefficients used in this study^19,30,42^. Hence, ignoring the influence of emission factors, the activity data of the OBB emission constructed based on multiple active fires in CHOBE was effectively reduce the underestimation by only using MODIS observations.

The hourly variations of GFEDv4.1 are similar to CHOBE, but there is 1~3 hours difference between its peak hour and CHOBE because its diurnal cycle was obtained based on historical GOES observations in the western hemisphere rather than real-time detections^16^. In comparison to CHOBE, FINNv1.5 and GFASv1.2 failed to capture the emission peaks around 15:00, whereas hourly emissions from FEERv1.0 approximately the CHOBE peaks, but did not capture the troughs at night.

**Fig. 6:**
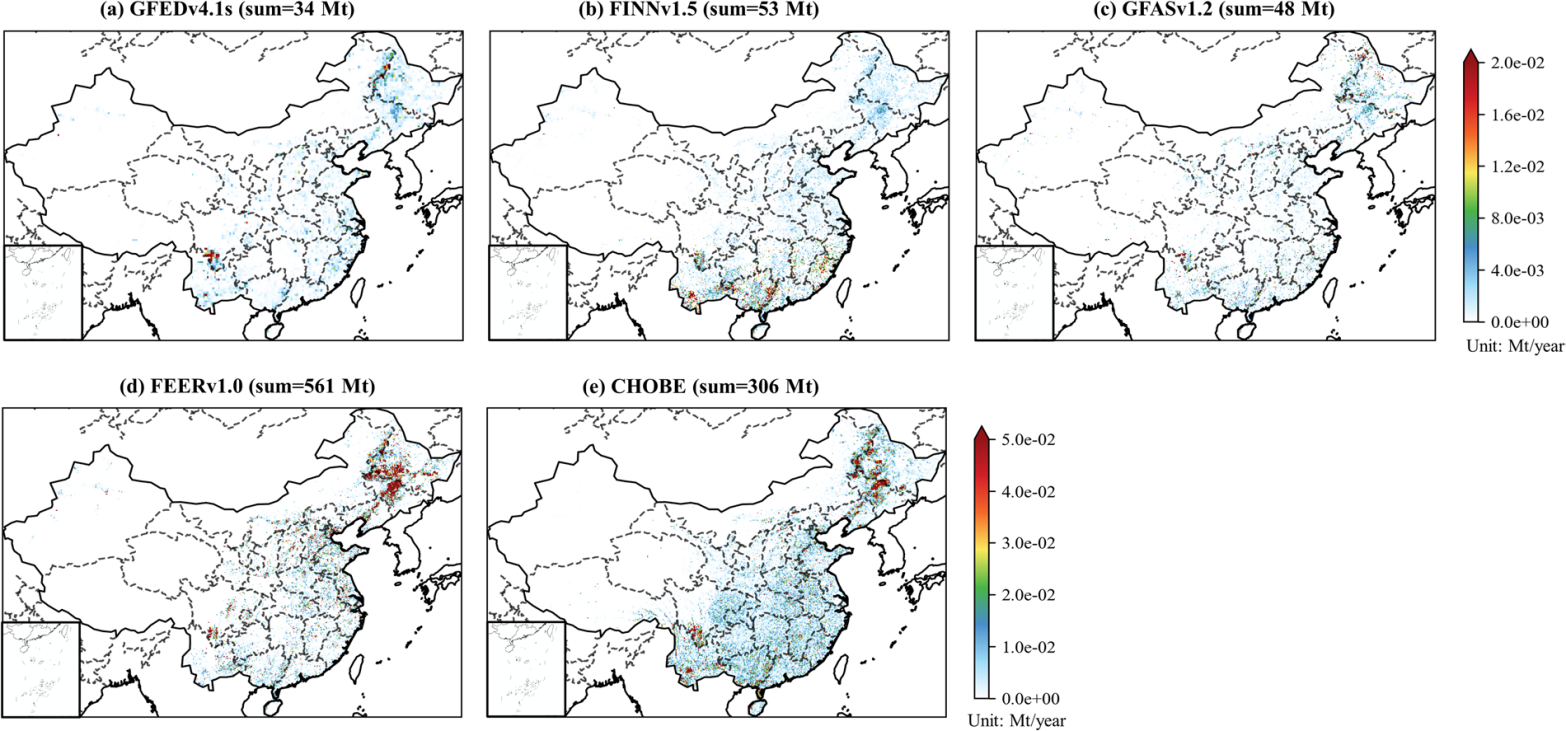
Spatial distributions of CO_2_ emissions from CHOBE and other datasets in mainland China, 2020. The horizontal resolution of all the datasets was converted to 0.1° to facilitate the spatial comparison.

Spatial distributions of CHOBE and existing datasets were displayed in Fig. 6 using CO_2_ emissions in 2020 as an illustration. All the datasets displayed comparable spatial patterns, with the northeast and south China being the main hot spots. CHOBE covered 2.35 × 10^4^ grids for a spatial resolution of 0.1°, whereas FINNv1.5, GFASv1.2 and FEERv1.0 covered ~ 1.71 × 10^4^ grids, indicating that CHOBE captured more burnings than existing datasets that relied solely on MODIS observations.

## Usage Notes

CHOBE contains gridded emissions of 6 air pollutants (CO, NOx, SO_2_, NH_3_, VOCs, PM_2.5_) and 3 greenhouse gases (CO_2_, CH_4_, N_2_O) from OBB during 2016 to 2020 in mainland China with a temporal resolution of 1 hour and spatial resolution of 2 km. CHOBE fills in the gap left by China’s lack of hourly OBB emission inventories when dealing with the considerable diurnal changes in the OBB. CHOBE can be used to investigate spatiotemporal variations and identify the driving forces behind OBB emissions during China’s 13th Five-Year Plan period, which included rigorous atmospheric control policies at the national, provincial, and city levels. By providing high-resolution OBB emission inputs, CHOBE is projected to increase modelling accuracy. CHOBE can also be applied to evaluate emission reductions and provide support for the formulation of precise OBB preventive and control strategies.

The hourly OBB emissions estimate in this study has three limitations: (1) Some minor fires that fell under the Himawari-8 AHI monitor restriction or occurred outside of the polar-orbiting satellite overpassing period were still missed. More satellite observations, such as VIIRS on board the National Oceanic and Atmospheric Administration-20 (NOAA-20) at the spatial resolution of 375 m and the Advanced Geosynchronous Radiation Imager (AGRI) onboard the Feng Yun-4A (FY4A) with a monitor frequency of 15 mins, can be fused to further improve the spatiotemporal representation of hourly OBB emissions. (2) Land-use was used to distinguish crop straw burning and forest fires, but crop straw burning and forest fires are easy to be confused as they are generally near to each other. Field investigations are recommended to ensure the accuracy of crop straw burning and forest fire identification. (3) Localized measurements of FRE-based crop straw burning and forest fire emission coefficients will be done in the future to reduce the uncertainties in OBB emission estimations since OBB emission coefficients varied in different regions.

### Supplementary information

Supplementary Information

## Data Availability

Sample codes for estimating gridded emissions from hourly FRE and regional OBB emission coefficients, named as “Code for emission estimation”, are available on the online platform figshare alongside the CHOBE datasets^38^.
