# Supplementary material for: Hourly emissions of air pollutants and greenhouse gases from open biomass burning in China during 2016–2020
Source: Sci Data. 2023 Sep 16;10:629. doi: 10.1038/s41597-023-02541-0 (PMC10505139; doi:10.1038/s41597-023-02541-0)
Supplement: Supplementary file 1 — Supplementary Information [file 41597_2023_2541_MOESM1_ESM.pdf]

Supplementary Information of

Hourly emissions of air pollutants and greenhouse gases from open biomass burning in China during 2016 - 2020

Yuanqian Xu[1], Zhijiong Huang[2], Jiashu Ye[3], Junyu Zheng[3*]

[1] College of Materials and Chemical Engineering, Zhengzhou University of Light Industry, Zhengzhou, 450001, China

[2] Institute for Environment and Climate Research, Jinan University, Guangzhou, 511443, China

[3] Sustainable Energy and Environment Thrust, The Hong Kong University of Science and Technology (Guangzhou), Guangzhou, 511453, China.

* Corresponding authors: Junyu Zheng (junyuzheng@hkust-gz.edu.cn)

Supplementary information

Table S1. Provinces in different in-field crop straw burning region......................3
Table S2. MODIS and Himawari-8 FRP modification factor in different region...4
Table S3. Provincial CO_2 emissions from 2016 to 2020 in Mt/year......................5
Table S4. Uncertainties of gridded daily FRE for each region. 6
Table S5. Comparisons of FRE-based OBB emission coefficients in different studies (unit: g/MJ). 7
Table S6. Uncertainties of annual OBB emissions in different region. 8
Figure S1. Spatial distributions of different forest types in mainland China........10
Figure S2. Regression relationships between burning duration (BD) and peak FRP for the 7 crop straw burning regions and 4 forest fire regions.............................11
Figure S3. FRP variations during burning cycles with different BD in the 7 crop straw burning regions and 4 forest fire regions observed by Himawari-8 AHI....12

Table S1. Provinces in different in-field crop straw burning region.

Region	Provinces
Region 1	Inner Mongolia, Liaoning, Jilin, Heilongjiang
Region 2	Beijing, Tianjin, Hebei, Shanxi
Region 3	Shandong, Henan, Hubei, Anhui
Region 4	Shanghai, Zhejiang, Jiangsu
Region 5	Fujian, Jiangxi, Hunan, Guangdong, Guangxi, Hainan
Region 6	Chongqing, Sichuan, Guizhou, Yunnan, Tibet
Region 7	Shaanxi, Gansu, Ningxia, Qinghai, Xinjiang

Table S2. MODIS and Himawari-8 FRP modification factor in different region.

Region	α_M	α_H
In-field crop straw burning Region 1	1.5710	1.1997
In-field crop straw burning Region 2	1.6832	1.2595
In-field crop straw burning Region 3	1.5585	1.2301
In-field crop straw burning Region 4	1.6751	1.3263
In-field crop straw burning Region 5	1.5933	1.2318
In-field crop straw burning Region 6	1.6550	1.2382
In-field crop straw burning Region 7	1.8185	1.2647
Evergreen broad-leaf forest fires	1.7345	1.3453
Mixed forest fires	1.8110	1.2836
Deciduous broad-leaf forest fires	1.4078	1.2612
Needle-leaf forest fires	1.8377	1.3109

Table S3. Provincial CO_2 emissions from 2016 to 2020 in Mt/year.

Province	2016	2017	2018	2019	2020
Beijing	0.42	0.37	0.26	0.26	0.27
Shanghai	0.12	0.10	0.11	0.13	0.11
Tianjin	1.82	1.44	0.82	1.15	1.13
Chongqing	4.11	3.45	3.23	4.59	3.68
Hebei	10.37	11.41	8.40	11.13	8.89
Shanxi	6.68	5.32	7.87	8.83	6.95
Inner Mongolia	14.42	11.75	10.04	10.62	15.91
Liaoning	10.10	12.93	6.49	10.57	9.50
Jilin	10.81	27.94	12.36	18.83	36.28
Heilongjiang	51.30	104.72	23.35	57.95	40.58
Jiangsu	4.31	4.01	3.81	4.36	4.07
Zhejiang	5.85	4.62	4.09	3.46	4.08
Anhui	9.83	10.26	9.80	13.00	9.27
Fujian	8.93	8.41	8.66	7.59	7.12
Jiangxi	22.63	17.97	15.82	12.45	9.70
Shandong	8.35	10.55	7.91	9.48	10.16
Henan	10.57	8.35	8.06	10.49	10.54
Hubei	17.54	12.80	13.08	15.98	9.57
Hunan	24.66	17.17	14.88	14.93	9.91
Guangdong	23.12	19.56	17.84	12.39	10.94
Guangxi	25.99	20.79	22.00	20.20	19.65
Hainan	1.98	1.94	2.09	2.01	1.74
Sichuan	16.02	14.88	13.47	15.82	25.06
Guizhou	10.61	7.81	11.00	9.14	6.45
Yunnan	36.78	25.00	25.64	32.75	30.30
Tibet	8.46	9.17	6.41	8.32	5.86
Shaanxi	5.24	3.37	4.15	5.17	4.41
Gansu	3.92	2.17	2.98	2.91	2.96
Ningxia	1.19	0.91	1.05	0.85	0.80
Qinghai	0.77	0.32	0.53	0.33	0.35
Xinjiang	0.09	0.04	0.03	0.02	0.02

Table S4. Uncertainties of gridded daily FRE for each region.

Region	Uncertainties
In-field crop straw burning Region 1	-24.28% ~ 43.82%
In-field crop straw burning Region 2	-21.86% ~ 45.03%
In-field crop straw burning Region 3	-26.68% ~ 43.91%
In-field crop straw burning Region 4	-66.51% ~ 77.51%
In-field crop straw burning Region 5	-59.10% ~ 71.29%
In-field crop straw burning Region 6	-34.24% ~ 48.03%
In-field crop straw burning Region 7	-22.55% ~ 39.85%
Evergreen broad-leaf forest fires	-27.90% ~ 46.32%
Mixed forest fires	-31.56% ~ 43.66%
Deciduous broad-leaf forest fires	-26.93% ~ 45.64%
Needle-leaf forest fires	-31.41% ~ 43.50%

Table S5. Comparisons of FRE-based OBB emission coefficients in different studies (unit: g/MJ).

Source	Achieving approach	CO_2	CO	NOx	NH_3	$PM_{2.5}$	BC+OC
This study	Regression models and coefficient conversion	620.74~1060.30	29.44~48.38	0.54~2.42	0.31~1.44	4.18~6.82	1.78~4.01
Freeborn et al. (2008) [1]	Laboratory measurement	652.26~1210.44	13.18~41.88	0.66~4.19		1.28~14.41	
Vermote et al. (2009) [2]	top-down constrained						2.7~14.4
Adams et al. (2019) [3]	top-down constrained		49	0.4	1.1~1.2		
Schreier et al (2014) [4]	top-down constrained			0.28~1.56			
Tanimoto et al. (2015) [5]	top-down constrained			1.73~12.62			
Andreas (2019) [6]	Unit conversion [a]	371.17~1071.31	12.49~105.49	0.16~2.29	0.03~1.73	0.57~14.7	0.18~7.16

[a] Emission factors were evaluated and integrated from over 370 published studies with the unit of g/kg by Andreas (2019) [6]. We used the conversion factor of 0.41g/MJ (Vermote et al (2009) [2]) to ensure the consistent unit of emission coefficient.

Table S6. Uncertainties of annual OBB emissions in different region.

Region	CO	NOx	SO_2	NH_3	VOCs	$PM_{2.5}$	CO_2	CH_4	N_2O
In-field crop straw burning Region 1	-71% ~ 71%	-50% ~ 50%	-86% ~ 86%	-62% ~ 62%	-103% ~ 103%	-53% ~ 53%	-16% ~ 16%	-102% ~ 102%	-42% ~ 42%
In-field crop straw burning Region 2	-71% ~ 71%	-50% ~ 50%	-86% ~ 86%	-62% ~ 62%	-103% ~ 103%	-53% ~ 54%	-16% ~ 16%	-102% ~ 103%	-42% ~ 42%
In-field crop straw burning Region 3	-71% ~ 71%	-50% ~ 50%	-86% ~ 87%	-62% ~ 62%	-104% ~ 103%	-52% ~ 53%	-16% ~ 16%	-102% ~ 101%	-42% ~ 42%
In-field crop straw burning Region 4	-72% ~ 77%	-51% ~ 57%	-87% ~ 92%	-63% ~ 69%	-102% ~ 108%	-54% ~ 59%	-25% ~ 27%	-102% ~ 107%	-44% ~ 49%
In-field crop straw burning Region 5	-71% ~ 73%	-50% ~ 52%	-87% ~ 88%	-62% ~ 64%	-103% ~ 105%	-53% ~ 55%	-19% ~ 20%	-103% ~ 104%	-43% ~ 44%
In-field crop straw burning Region 6	-71% ~ 71%	-50% ~ 50%	-86% ~ 86%	-62% ~ 62%	-102% ~ 102%	-53% ~ 53%	-16% ~ 16%	-101% ~ 102%	-42% ~ 42%
In-field crop straw burning Region 7	-71% ~ 72%	-50% ~ 50%	-86% ~ 87%	-62% ~ 63%	-103% ~ 103%	-53% ~ 53%	-16% ~ 16%	-102% ~ 102%	-42% ~ 42%

Evergreen broad-leaf forest fires	-43% ~ 44%	-58% ~ 58%	-66% ~ 67%	-69% ~ 69%	-86% ~ 85%	-76% ~ 76%	-8% ~ 8%	-52% ~ 52%	-45% ~ 45%
Mixed forest fires	-44% ~ 44%	-58% ~ 58%	-67% ~ 67%	-69% ~ 69%	-86% ~ 86%	-77% ~ 77%	-8% ~ 8%	-52% ~ 52%	-45% ~ 45%
Deciduous broad-leaf forest fires	-44% ~ 43%	-58% ~ 58%	-67% ~ 67%	-69% ~ 69%	-85% ~ 86%	-76% ~ 77%	-8% ~ 8%	-52% ~ 52%	-45% ~ 45%
Needle-leaf forest fires	-44% ~ 44%	-58% ~ 58%	-67% ~ 67%	-69% ~ 70%	-86% ~ 86%	-76% ~ 76%	-9% ~ 9%	-52% ~ 52%	-45% ~ 45%

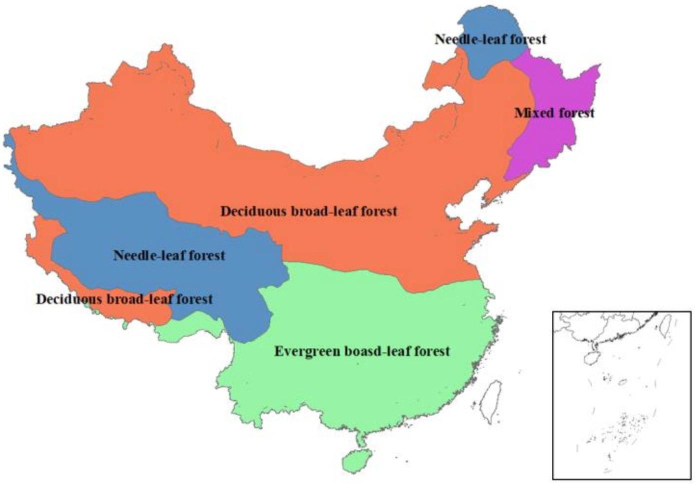

Figure S1. Spatial distributions of different forest types in mainland China. (The spatial distributions of forest types were reclassified on the base of the vegetation regionalization data available on the Resource and Environment Science and Data Center https://www.resdc.cn/Default.aspx).

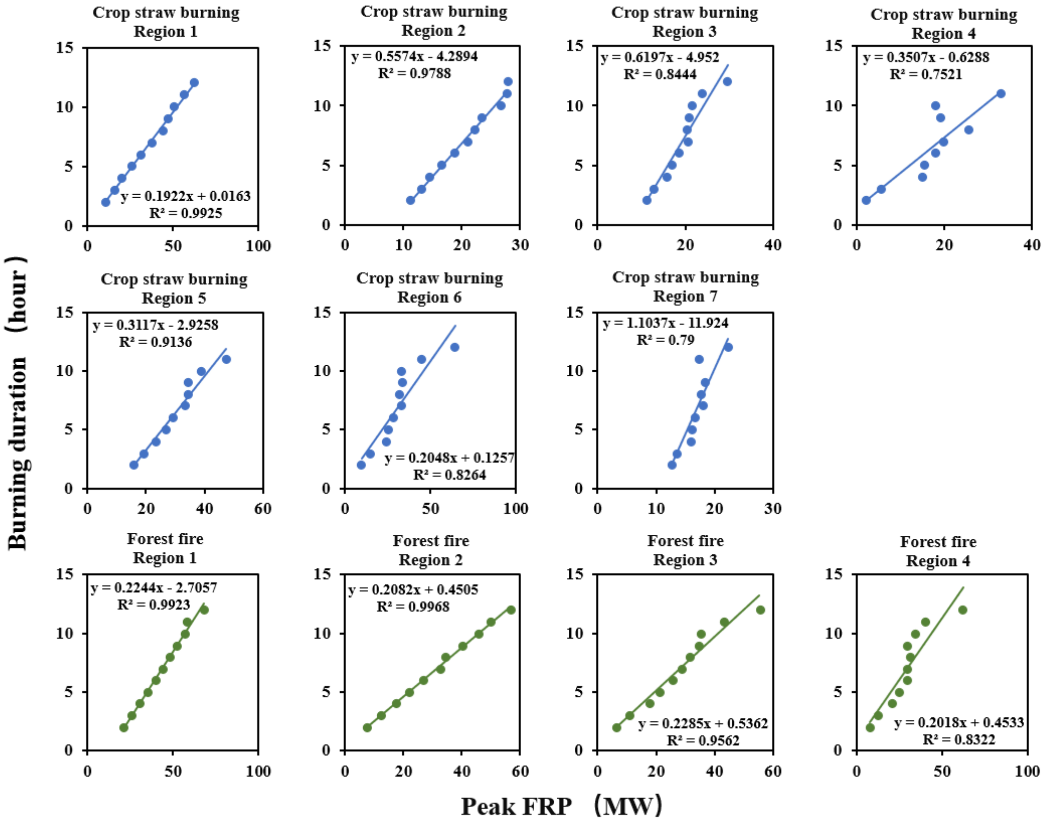

Figure S2. Regression relationships between burning duration (BD) and peak FRP for the 7 crop straw burning regions and 4 forest fire regions.

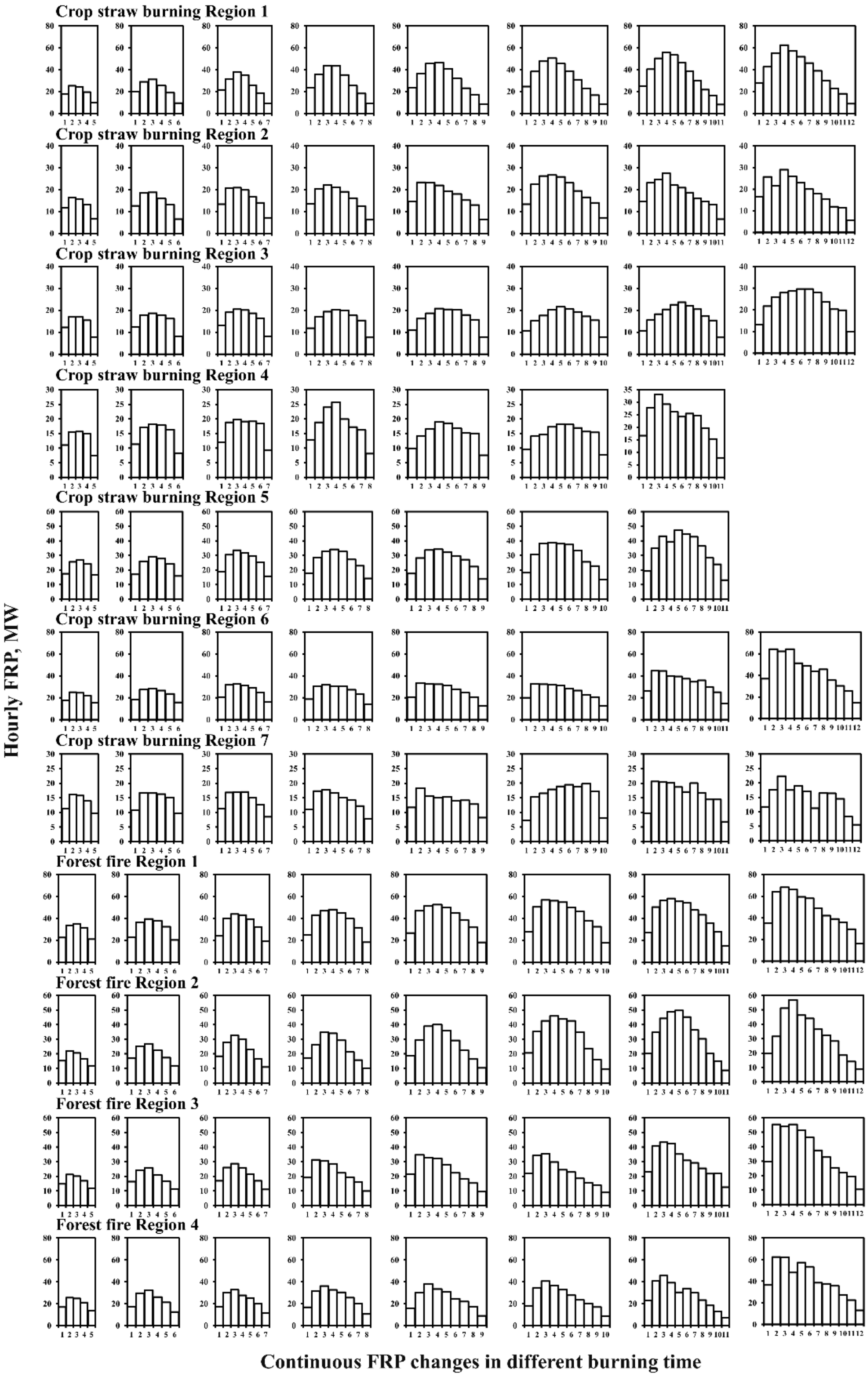

Figure S3. FRP variations during burning cycles with different BD in the 7 crop straw burning regions and 4 forest fire regions observed by Himawari-8 AHI.

References

1. Freeborn, P. *et al*. Relationships between energy release, fuel mass loss, and trace gas and aerosol emissions during laboratory biomass fires. *J. Geophys. Res. Atmosph*. **113**, D1 (2008).
2. Vermote, E. *et al*. An approach to estimate global biomass burning emissions of organic and black carbon from MODIS fire radiative power. *J. Geophys. Res. Atmosph*. **114**, D18 (2009).
3. Adams, C. *et al*. Satellite-derived emissions of carbon monoxide, ammonia, and nitrogen dioxide from the 2016 Horse River wildfire in the Fort McMurray area. *Atmos. Chem. Phys*. **19**, 2577-2599 (2019).
4. Schreier, S. *et al*. The empirical relationship between satellite-derived tropospheric NO2 and fire radiative power and possible implications for fire emission rates of NOx. *Atmos. Chem. Phys*. **14**, 2447-2466 (2014).
5. Tanimoto, H., Ikeda, K., Boersma, K., Van Der, A. & Garivait, S. Interannual variability of nitrogen oxides emissions from boreal fires in Siberia and Alaska during 1996–2011 as observed from space. *Environ. Res. Lett*. **10**, 065004 (2015).
6. Andreae, M. Emission of trace gases and aerosols from biomass burning–an updated assessment. *Atmos. Chem. Phys*. **19**, 8523-8546 (2019).
